# O8-1 24-Hour Movement Behavior and Fundamental Movement Skills in Pre-School Children: A Compositional Analysis

**DOI:** 10.1093/eurpub/ckac094.057

**Published:** 2022-08-29

**Authors:** Clarice Lucena Martins, Cain Craig Truman Clark, Jéssica Gomes Mota, Thaynã Alves Bezerra, Luís Filipe Gomes Lemos, Cézane Priscilla Reuter, Michael Dunca, Jorge Mota

**Affiliations:** 1 Department of Physical Education, Federal University of Paraiba, João Pessoa, Brazil; 2 Coventry University, Research Associate School of Health Life Sciences, Coventry, United Kingdom; 3 Department of health Sciences, Santa Cruz do Sul University, Santa Cruz do Sul, Brazil; 4 Centre for Applied Biological and Exercise Sciences, Coventry University, Coventry, United Kingdom; 5 CIAFEL, FADEUP, Porto, Portugal

**Keywords:** pre-schoolers, motor competence, physical activity, accelerometers, 24h movement guidelines

## Abstract

**Background:**

Studies that have analyzed the association between the different movement behaviors and fundamental movement skills (FMS), have considered it in an independent manner, disregarding the compositional nature of 24-hour movement behaviors (24h MB). The aim of this study was to investigate relationships between the 24h MB and FMS in low-income preschoolers.

**Methods:**

Two hundred and four preschoolers of both sexes (4.5±0.8 years old; 101boys) provided objectively assessed physical activity (PA) and sedentary time (ST) data (Actigraph wGT3X), and FMS assessments (TGMD-2). Sleep duration (*SD*) was reported by parents through interview. Association of daily time composition of movement behaviors with FMS was explored using compositional data analysis (R Core Team, 3.6.1).

**Results:**

Our data highlighted that no single movement behaviour significantly predicted locomotor, manipulative, or total motor competence. When data were considered as a 24h MB composition based on PA, ST and SD, adjusted for age, BMI and sex, the composition significantly predicted locomotor score (P > 0.0001; r2 = 0.31), manipulative score (P > 0.0001; r2 = 0.19) and total motor competence score (P > 0.0001; r2 = 0.35), respectively.

**Conclusion:**

Our study suggests that the 24h MB composition is more important for adequate FMS then any individual, movement behavior. This represents an important finding, particularly for creating and optimizing interventions to benefit child health.

